# Commentary: But Is It really Art? The Classification of Images as “Art”/“Not Art” and Correlation with Appraisal and Viewer Interpersonal Differences

**DOI:** 10.3389/fpsyg.2017.02328

**Published:** 2018-01-08

**Authors:** Marcos Nadal, Víctor Gallardo, Gisèle Marty

**Affiliations:** ^1^Human Evolution and Cognition Group-CSIC, University of the Balearic Islands, Palma, Spain; ^2^Psychology, University of the Balearic Islands, Palma, Spain; ^3^Hospital Can Misses, Ibiza, Spain

**Keywords:** art, empirical aesthetics, formalism, constructionism, cognition

Scientific aesthetics aims to understand the creation and appreciation of aesthetics and art using scientific methods devised to yield valid and reliable empirical evidence. The pursuit of such evidence, as in other domains of science, has made of the laboratory an indispensable research environment, and of laboratory experimental procedures the preferred means to conduct research (Fechner, 1876; Külpe, 1907; Valentine, 1913). In the laboratory, reproductions of artworks have often been used as stimuli. Sometimes because the object of study is the experience of these as artworks, other times because the object of study is an aesthetic dimension (e.g., balance) along which they vary systematically (Pearce et al., 2016). In both cases it is assumed that the artistic status of the stimuli is relevant to participants' responses, and that participants actually do regard those stimuli as artworks.

Pelowski et al. (2017) recently conducted an experiment to examine the validity of this key—but previously unquestioned—assumption. They asked participants to classify images of a broad range of artworks as either works of art or not works art, and also to express their liking for them. Their main results show that participants often did not classify the depicted objects as artworks. For instance, participants did not believe abstract paintings were artworks on 75% of trials, and did not believe readymade sculptures were artworks on more than 50% of trials. These results call into question the assumption that participants will necessarily regard artistic stimuli presented in an experimental setting as actual artworks. The implication is important, because, as Pelowski et al. (2017) showed, the attributed artistic status greatly influenced liking: liking ratings for images classified as art were about 20% higher than for images classified as not art.

We believe, however, that Pelowski et al.s' (2017) study has even deeper implications. Not only do its results challenge a methodological assumption guiding experimental work in the laboratory; they challenge the deeply rooted conception of aesthetic experience as a response triggered by formal features of objects (Silvia, 2012; Vartanian, 2014), particularly combinations and arrangements of such elements as lines, colors, or shapes, among others. Scientific aesthetics has been committed to this formalist understanding of aesthetic experience since the start (Bosanquet, 1904). Fechner (1876) developed experimental aesthetics by using Psychophysics' methods for scaling sensory magnitude in relation to stimulation to transform Herbart's formalist speculations on the elementary relations among forms he believed governed pleasing and displeasing aesthetic impressions into an empirical program (Külpe, 1897). The main aim of this program was “to relate preferences to properties of the works of art or other objects that are presented” (Berlyne, 1971, p. 12), and at its core was the “conjecture that the aesthetic feeling originates in a *relation of the perceived impression to the reproduction* which it excites” (Külpe, 1895, p. 252). The truth of this assumption seemed so obvious it was embraced without question, first by the early experimental aestheticians (e.g., Major, 1895; Pierce, 1896; Angier, 1903; MacDougall, 1903; Pfuffer, 1903; Martin, 1906; Valentine, 1913), and then by behaviorists, who saw the arrow linking “formal feature” to “aesthetic impression” as a specific instance of the all-explaining “stimulus-response” scheme (Beebe-Center, 1932). This assumption also appealed to later information-processing approaches to art and aesthetics (Moles, 1966; Berlyne, 1971; Pickford, 1972), because it neatly matched the general input-system-output blueprint of cognition. This is how the conception of the experience of art as a response to object features burrowed its way under the surface of scientific aesthetics, all the way from its beginning to this very day.

Pelowski et al. (2017) deliver decisive evidence showing that the experience of art cannot be conceived as a response to a presented object, as in a psychophysical experiment where participants are passively subjected to stimulation. People actively participate in the construction of their own experience of art. To this experience they contribute their knowledge and interests (Cupchik and Gebotys, 1988; Kirk et al., 2009a), motivations and expectations (Smith and Wolf, 1996; Kirk et al., 2009b), affect and emotion (Nadal and Rosselló, 2015), movements and exploration (Brieber et al., 2014, 2015), and ideas and beliefs about what art is and what it looks like (Pelowski et al., 2017). The experience of art is not brought about by a presented object; it begins before any object is presented, with the seeking, expecting, and anticipating the object. This flurry of knowledge, expectations, and beliefs prior to and during the encounter with an object is what actually shapes it into an experience of art.

In short, experiencing art is not merely a matter of undergoing, but also of acting (Dewey, 1934). The experience of art, however, is not special in this regard. The construction of experience is common to all domains of cognition, including perception (“Whether beautiful or ugly or just conveniently at hand, the world of experience is produced by the [person] who experiences it,” Neisser, 1967, p. 3), memory (“remembering appears to be far more decisively an affair of construction rather than one of mere reproduction,” Bartlett, 1932, p. 205), and emotion (“each emotional episode is constructed rather than triggered,” Barrett and Russell, 2015, p. 4). Indeed, cognitive psychology originally intended to “discover and to describe formally the meanings that human beings created out of their encounters with the world, and then to propose hypotheses about what meaning-making processes were implicated (Bruner, 1990, p. 2).

All of the above does not entail the rejection of the laboratory context and methods for research on art. Laboratory work is not an end in itself; it is a means to an end. What is needed from scientific aesthetics now is not so much a change in means as in ends. It needs to shift its primary purpose from studying how formal features of art and other objects elicit preferences to explaining the psychological and neural mechanisms underlying people's active creation of meaningful art and aesthetic experiences.

## Author contributions

MN and VG conceived the research. MN, VG, and GM wrote the paper together.

### Conflict of interest statement

The authors declare that the research was conducted in the absence of any commercial or financial relationships that could be construed as a potential conflict of interest.
